# Whole blood profiling of leprosy type 1(reversal) reactions highlights prominence of innate immune response genes

**DOI:** 10.1186/s12879-018-3348-6

**Published:** 2018-08-24

**Authors:** Jamile Leão Rêgo, Nadja de Lima Santana, Paulo Roberto Lima Machado, Marcelo Ribeiro-Alves, Thiago Gomes de Toledo-Pinto, Léa Cristina Castellucci, Milton Ozório Moraes

**Affiliations:** 1 Instituto Nacional de Ciência e Tecnologia em Doenças Tropicais, Salvador, Brazil; 2 Programa de Pós-graduação em Ciências da Saúde da Universidade Federal da Bahia, Salvador, Brazil; 3 Fundação Oswaldo Cruz-FIOCRUZ, Rio de Janeiro, Brazil; 4 Hospital Universitário Professor Edgard Santos, Serviço de Imunologia, Rua João das Botas, S/N°, 5° andar, Salvador, Bahia 40.110-160 Brazil

**Keywords:** Leprosy reactions; gene expression; profile, Parkin, Pro-inflammatory, Type-I IFN, OASL

## Abstract

**Background:**

The major factors contributing for nerve damage and permanent disabilities in leprosy are type 1 or reversal reactions (RR) and type 2 or erythema nodosum leprosum (ENL). Gene profiling of leprosy reactions have shown that different pathways are activated during the course of reactions, which is consistent with the exacerbated immune response exhibited by these patients.

**Methods:**

We used qPCR to screen a panel of 90 genes related to the immune response in leprosy in RNA-derived peripheral leukocytes of patients with (*N* = 94) and without leprosy reactions (*N* = 57) in order to define expression signatures correlated to RR or ENL.

**Results:**

Our results show that there is a marked signature for RR in the blood, comprising genes mostly related to the innate immune responses, including type I IFN components, autophagy, parkins and Toll like receptors. On the other hand, only Parkin was differentially expressed in the ENL group.

**Conclusions:**

The data put together corroborates previous work that brings evidence that an acute uncontrolled exacerbated immune response designed to contain the spread of M. leprae antigens might be cause of RR pathogenesis. Identifying a blood profile useful to predict leprosy reactions prior to its development might help to reduce the morbidity associated to this disabling disease.

## Background

Leprosy is a chronic infectious disease caused by *Mycobacterium leprae*. The bacilli invades Schwann cells and macrophages of the skin leading the tissue injury, which is the major reason for its pathogenesis [1–3]. Leprosy presents a wide variety of clinical presentations, including the indeterminate (I), tuberculoid (TT), borderline (BT, BB, BL) and lepromatous (LL) forms. In addition, about 20–50% of leprosy patients, depending on the population studied, can be affected by acute inflammatory episodes known as leprosy reactions, as so called type 1 (Reversal Reaction) or type 2 (Erythema Nodosum Leprosum- ENL) [4, 5]. Either RR or ENL are observed in all borderline forms prior, during or after completion of multidrug therapy. RR involves the active participation of T lymphocytes and abrupt episodes of intense local delayed-type hypersensitivity to *M. leprae* in skin and/or nerves. On the other hand, ENL is typical of the BL and LL forms and is correlated to a systemic reaction involving a cytokine storm and also deposition of immune complexes in skin and organs [6, 7]. Regardless its type, leprosy reactions are an important contributing factor of nerve damage among patients with leprosy. The identification of host-derived biomarkers correlated to leprosy reactions might point out new tests to predict increased risk of developing the occurrence of reactional episodes thus helping to prevent its irreversible sequels. There are only a few transcriptomic studies searching for genes related to the development of leprosy reactions. Among these, a role for pro and anti-inflammatory regulators, IFN-induced genes, complement components, among others have been described [5, 6, 8].

## Methods

**Fig. 1:**
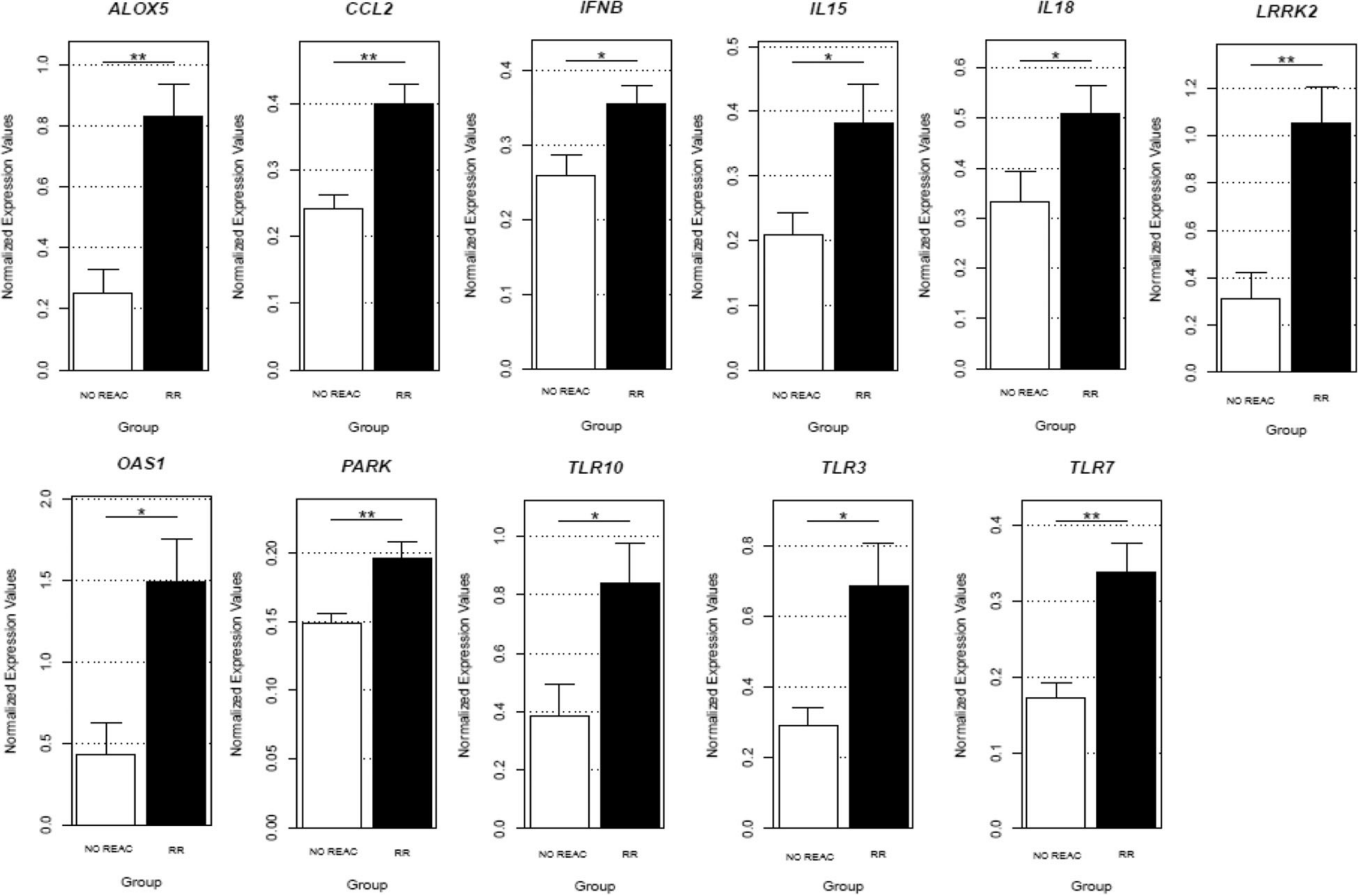
Gene expression of whole blood leukocytes in patients with RR as compared to patients without leprosy reactions. A group of genes differentially expressed in RR samples was identified, consisting of 1 chemokine gene (*CCL2* logFC = 4.04), 2 PARK genes (*PARK* logFC =3.27, *LRRK2* logFC =3.09), 1 eicosanoid metabolism gene (*ALOX5* logFC =2.99), 4 pattern recognition receptor genes (Toll-like receptors -TLRs: *TLR7* logFC =2.98, *TLR10* logFC =3.07, *TLR3* logFC =2.74, and C-lectin receptors-CLRs: *CLEC5A* logFC =2.81), 2 cytokine genes (*IL18* logFC =2.34, *IL15* logFC = 2.42) and 2 type I IFN genes (*IFNB* logFC =2.36, *OAS1* logFC = 2.51)

In this study we analyzed the expression of a panel of relevant immune response genes in RNA-derived from peripheral blood leukocytes of leprosy patients with and without reactions in order to identify gene expression signatures associated with either RR or ENL. One hundred and fifty one cDNA samples of patients divided into three groups were used: 57 patients with no evidence of reactions (hereinafter referred to as No Reaction Group - NR), 50 patients with RR and 44 patients with ENL. Subjects were diagnosed according to the Brazilian’s Ministry of Health guidelines in the leprosy outpatient clinics from Hospital Universitário Professor Edgard Santos and Hospital Couto Maia in the city of Salvador-Bahia, Brazil. Patients were classified according to a Ridley–Jopling classification and by the WHO field classification [9, 10], as previously reported for studies of patients recruited from this hospital in Salvador [11]. Detailed complementary data about the participants are described in Table 1. Written informed consent was obtained from all patients after approval of the study by the Ethics Committee from the Federal University of Bahia (number 891.963). Peripheral leukocytes from patients free of immunosuppressants such as thalidomide or prednisone were homogenized in TRIzol (Thermo Fisher Scientific). RNA was extracted using the PureLink ™ RNA Mini Kit (Thermo Fisher Scientific) and the total RNA concentration was determined in optical density spectrophotometer (260 and 280 nm). The cDNA conversion was performed using the High Capacity cDNA Reversion Transcription Kit (Applied Biosystems) following the manufacturer’s instructions. The expression of 90 target genes and 4 normalizing genes was performed by medium- throughput quantitative q-PCR using the microfluidic system Biomark (Fluidigm, CA). The analysis was performed from the real-time fluorescence accumulation data of each sample (ΔRn), using the logistic function adjustment of four parameters to represent each amplification curve by the library of qpcR (R Development Core Team, 2009) version 2.922. Results: After filtering by QC, 35 genes were excluded and 55 analyzed. We first compared the paucibacillary (PB) versus multibacillary (MB) leprosy within the unreactional (NR) group checking for differences regarding these two disease poles. This analysis did not show any significant differences (*p* < 0.05, data not shown). Nevertheless, there was a differential pattern of gene expression between the NR and RR group as shown in Table 2. A set of genes belonging to different pathways that includes the *parkin* pathway, pattern recognition receptors (PRRs), type I IFNs precursors, inflammatory cytokines and chemokines and eicosanoid metabolism were significantly more expressed in RR patients as compared to the NR group (Fig. 1). This peculiar inflammatory signature for type 1 reaction has been described in previous works [5, 8] that also underpinned a mixed immune activation that seems to lead to the RR pathogenesis. On the other hand, only *PARK2* was significantly more expressed in leucocytes of ENL compared to unreactional patients (logFC =2.13 e *p* = 0.04), as well as TLR7 between RR and ENL subjects (logFC = − 2.72 e *p* = 0.02).

**Table 1:** Demographic and clinical characteristics of leprosy patients

A - Characteristics of the sample^a^				
	N individuals	Age, years ± SD	Gender M:F	
Cases / Reaction	94	44.05 (13.31)	58:36	
Controls / No Reaction	57	45.21 (14.98)	28:29	
B - Clinical characteristics of the cohort^a^				
*Clinical phenotype*			*n*	*(%)*
Tuberculoid (TT)			20	(13)
Borderline tuberculoid (BT)			30	(20)
Borderline (BB)			18	(12)
Borderline lepromatous (BL)			17	(11)
Lepromatous (LL)			49	(32)
Indeterminate leprosy (I)			11	(7)
Other forms (Neural)			6	(4)
*Total#*			*151*	*(100)*
C - Patients with reaction episode^a^				
RR			50	(53)
ENL			44	(47)
*Total#*			*94*	*(62)*
D - Patients without reaction episode^a^				
PB			47	(82)
MB			10	(18)
*Total#*			*57*	*(38)*

^a^Results are shown as N(%). Abbreviations: *SD* Standart deviation, *M* male, *F* female, *PB* paucibacillary, *MB* multibacillary, *RR* reversal reaction, *ENL* erythema nodosum leprosum. Patients were also classified under leprosy clinical spectrum according to Ridley & Jopling [9]

**Table 2:** Normalized gene expression values of whole blood leukocytes samples of leprosy patients with reactions (*n* = 94) and leprosy patients without reactions (*n* = 57)

Reaction vs No Reaction			
Gene	Description	log fold change	*p*.value*
CCL2	C-C Motif Chemokine Ligand 2	4.04	0.0016
PARK	parkinson protein 2, E3 ubiquitin protein ligase	3.27	0.0036
ALOX5	Arachidonate 5-Lipoxygenase	2.99	0.0108
TLR7	Toll Like Receptor 7	2.98	0.011
LRRK2	Leucine Rich Repeat Kinase 2	3.09	0.0161
IFNB	interferon beta 1	2.36	0.0228
TLR10	Toll Like Receptor 10	3.07	0.0236
IL18	Interleukin 18	2.34	0.0326
TLR3	Toll Like Receptor 3	2.74	0.0338
CLEC5A	C-Type Lectin Domain Containing 5A	2.81	0.0446
RR vs No Reaction			
CCL2	C-C Motif Chemokine Ligand 2	4.31	0.0002
TLR7	Toll Like Receptor 7	3.81	0.0012
PARK	parkinson protein 2, E3 ubiquitin protein ligase	3.16	0.002
ALOX5	Arachidonate 5-Lipoxygenase	3.32	0.0044
LRRK2	Leucine Rich Repeat Kinase 2	3.28	0.0056
IFNB	interferon beta 1	2.64	0.0104
TLR10	Toll Like Receptor 10	3.08	0.0122
TLR3	Toll Like Receptor 3	3.04	0.0122
IL18	Interleukin 18	2.65	0.014
OAS1	2′-5′-oligoadenylate synthetase 1	2.51	0.0376
IL15	Interleukin 15	2.42	0.0446
ENL vs No Reaction			
PARK	parkinson protein 2, E3 ubiquitin protein ligase	2.13	0.0436
ENL vs RR			
TLR7	Toll Like Receptor 7	−2.72	0.0214

^*^The genes were defined as differentially expressed by the criterion of *p*-value adjusted for multiple comparisons. Bayesian statistical analysis used a log fold change cutoff of > 1 and adjusted *p* value of < 0.05. - Abbreviations: *RR* reversal reaction, *ENL* erythema nodosum leprosum

## Discussion

Our RR signature corroborates data showing that *M. leprae* components and host cell destruction continue to stimulate the immune response in a sudden and acute manner during RR. Most pathogen-associated molecular patterns (PAMPs) and damage-associated molecular pattern molecules (DAMPs) bind specific PRRs such as Toll-like receptors and NOD-like receptors to orchestrate both, autophagy and IFN signaling [12, 13]. We hypothesized that the continued binding of PAMPs and DAMPs to TLRs caused by the pathogen components after killing destruction provides the necessary trigger for maintenance of the inflammatory process. The stimulation of innate mechanisms that comprise genes with autophagic activities such as *PARK* and *LRRK2*, in addition to the type I IFNs in the beginning of the process seems to be activated in order to clear killed mycobacteria, but it is unbalanced and exacerbated. Regarding the IFNs, the genes IFNB and *OAS1* (2–5 ‘oligoadenylate synthetase-1 gene) had a greater expression in RR samples. *OASL* was also shown to be upregulated in *M. leprae*–infected human macrophage cell lineages, primary monocytes, and skin lesion from patients with a disseminated form of leprosy; whereas *OASL* knock down was associated with decreased viability of *M. leprae* and upregulation of autophagy levels [14]. Additionally, the chemokine *CCL2* was the most expressed gene in our RR group. Recent reports have linked the STING signaling, type I IFN and CCL2 activation [14, 15]. During mycobacterial infection, this chemokine can be produced in a STING-dependent manner and it is actively involved in the recruitment of monocytes to the infection site [15] and also related to mycobacterial survival within macrophages [14]. Other works have pointed the participation of CCL2 in the pathogenesis of several inflammatory disorders such as atherosclerosis and autoimmune diseases [16–18]. Here, we could speculate that a prominent triggering of STING signaling and high expression of type I IFN and CCL2 may contribute to the attraction of immune cells and enhancement of inflammatory response during leprosy reaction.

Type 1 reaction or RR is caused by an amplified immune response possibly triggered by fragmented bacillary antigens available in the cell medium [19]. The main issue however, is that a dysregulated process of gene activation, aiming to contain the progress of *M. leprae* and eliminate the infection, will lead to the nerve and tissue damage. Indeed, persons with history of RR can keep an altered response to *M. leprae* antigens that differs from patients with unreactional leprosy for years after resolution of RR [8]. Additionally, our results show that the expression of *TLR3, TLR7* and *TLR10* were significantly increased in the reactions per se as well as in RR with *TLR7* and *TLR10* corroborating with data that fragments of bacterial destruction may be giving continuity to the characteristic inflammatory process of both reactional episodes in leprosy. On the other hand, ENL is characterized by a systemic inflammatory reaction. In this case, it might be possible that other set of genes related to the humoral immune response would be more active in these leucocytes. We need to expand our panel in order to identify which profile explains ENL.

## Conclusion

Overall our data strength previous data and reinforces a signature for RR that could help to guide future studies for developing tools to predict this condition among leprosy patients. Personalizing the treatment of individuals susceptible to the development of reactions will help increase the effectiveness of treatment and reduce morbidity and disability in leprosy.
